# Supplementary material for: Reliable repurposing of the antibody interactome inside the cell
Source: Nat Commun. 2026 Jan 31;17:2222. doi: 10.1038/s41467-026-69057-0 (PMC12963631; doi:10.1038/s41467-026-69057-0)
Supplement: Supplementary file 1 — Supplementary Information [file 41467_2026_69057_MOESM1_ESM.pdf]

Supplementary Information

Reliable repurposing of antibody interactome inside the cell

Caitlin M. O'Shea[1], Rushba Shahzad[1], Kimia Aghasoleimani[1], Stuart Newman[1], Jiraporn Panmanee[2], Leonard C. Schalkwyk[1], Greg N. Brooke[1], Fiona E. Benson[4], James S. Trimmer[5], Daryl A. Bosco[6], Takao Fujisawa[7,8], Hidenori Ichijo[7,8], Neil R. Cashman[9], Stanislav Engel[10], Gareth S. A. Wright[1]*

Supplementary Figures

Supplementary Figure 1. Physicochemical properties of complete and cytoplasmic human proteomes in comparison with antibody variable domains.

Supplementary Figure 2. Comparison of the relationship between experimentally defined solubility for forty-five scFv intrabodies and predicted solubility by nine solubility predictors

Supplementary Figure 3. Impact of variable heavy domain charge on scFv intrabody solubility.

Supplementary Tables

Supplementary Table 1. Coefficient of determination (r^2) for predicted physicochemical and biophysical properties of scFv molecules broken down into regions of the molecule with experimentally determined solubility.

Supplementary Table 2. Comparison of variable domain CDR and framework mean charge of 8993 antibody-coding memory B cells[16,67–72] using IMGT, Chothia and Kabat amino acid numbering schemes.

Supplementary Resources

Supplementary Data 1 – Sequence, description, predicted physicochemical characteristics and solubility of forty-five scFv intrabodies with various tags, linkers and domain orientations.

Supplementary Data 2 – 3B5H10-derived scFv intrabodies including sequence, construct ID, fusion tags, linker, net charge and ProteinMPNN$_{SOL}$ inverse folding settings**.**

Supplementary Data 3 – Sequence and description of 672 non-redundant scFv intrabodies targeting cytoplasmic proteins with native and inverse folded sequences.

Supplementary Data 4 – Anti-p53, UCHL1 and GFP scFv intrabody sequences with reference.

Supplementary Data 5 – Anti-SOD1 conformational specific scFv intrabodies with sequence and references.

Supplementary Data 6 – Anti-α-synuclein state specific scFv intrabodies with sequences and references.

Supplementary Software 1 – scFvright source code.

Supplementary Figures

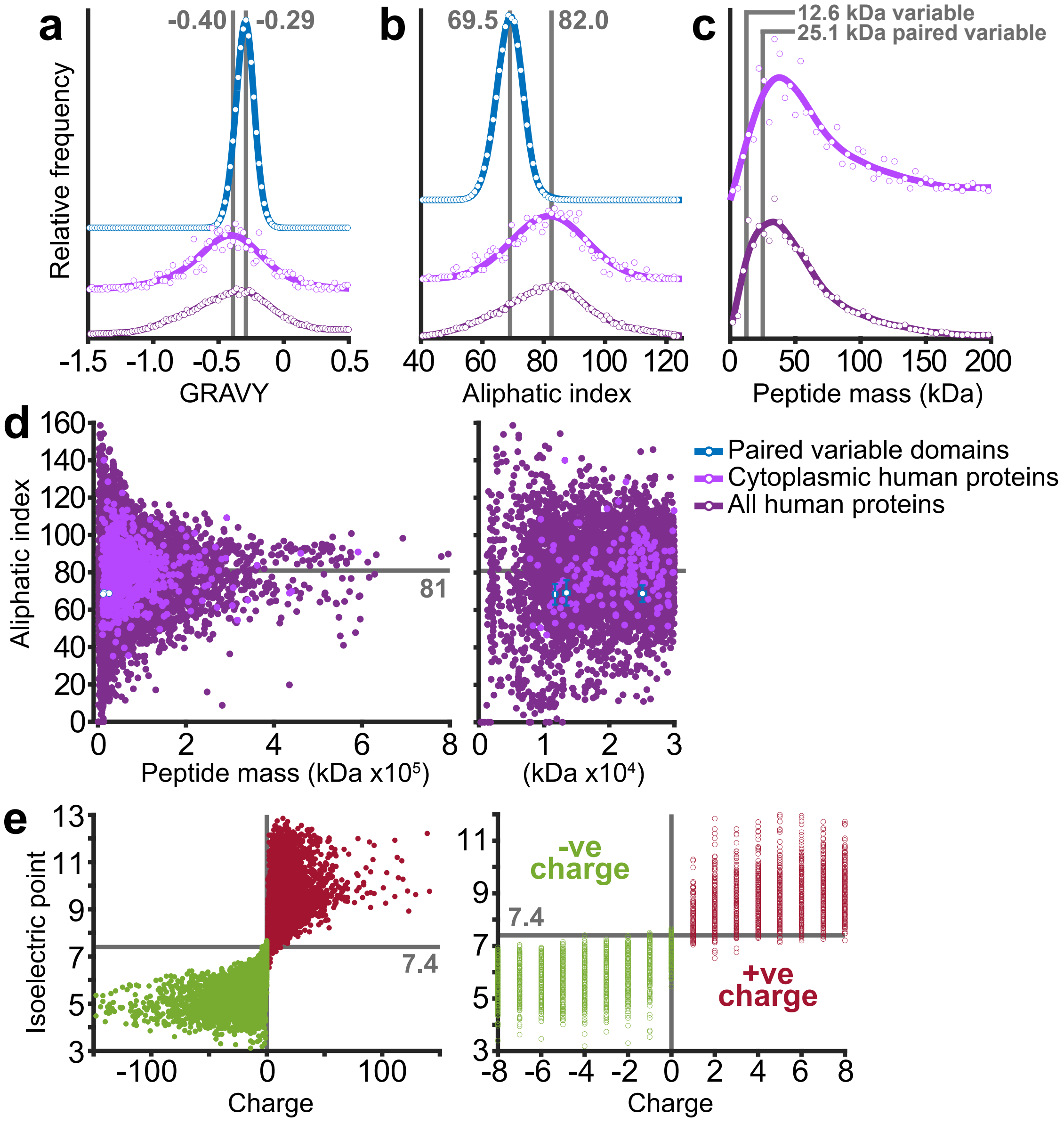

Supplementary Figure 1. Physicochemical properties of complete and cytoplasmic human proteomes in comparison with antibody variable domains. a. Fv grand average of hydropathy (GRAVY) and **b.** Aliphatic index. Modes distinguishing Fv and cytoplasmic proteins derived from the peak of the fitted curve are shown in grey. **c.** Molecular mass of single and paired variable domains in comparison with total and cytoplasmic human proteomes. **d.** Comparison of peptide mass and aliphatic index showing single variable and paired domains occupy a region within the main body of total and cytoplasmic human proteins synonymous with folded and stable proteins. Mean aliphatic index for total (80.9 ± 18.1) and cytoplasmic (81.2 ± 13.6), highlighted with grey line, can be compared with single VH (69.2 ± 6.7), VL (68.4 ± 5.5) and paired VLVH (68.8 ± 4.4). Error bars show one standard deviation. **e.** Relationship between net charge and isoelectric point across the human proteome, R^2 0.42. Data in **a**, **b** and **c** are normalised to area under the curve.

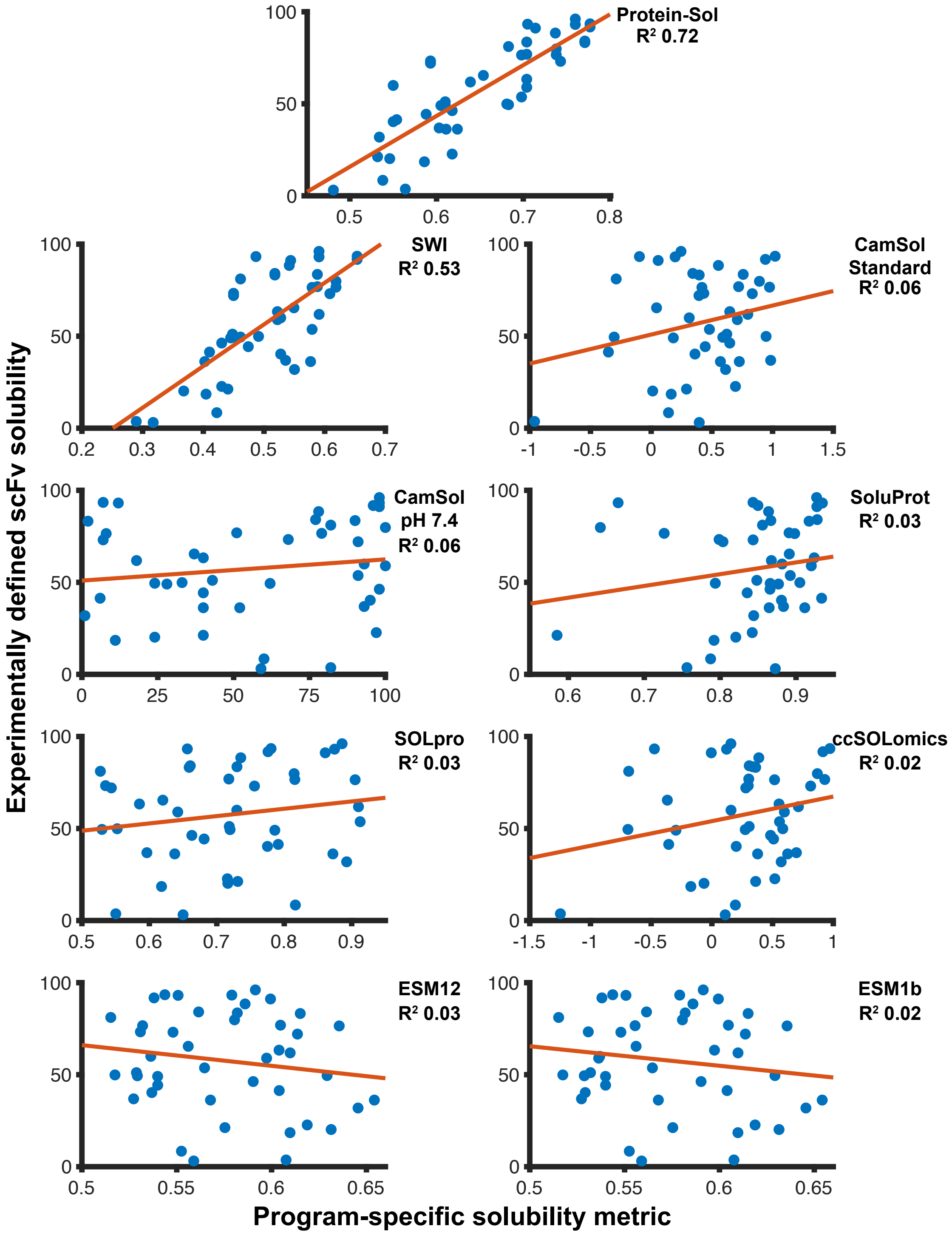

Supplementary Figure 2. Comparison of the relationship between experimentally defined solubility for forty-five scFv intrabodies and predicted solubility by nine solubility predictors[63].

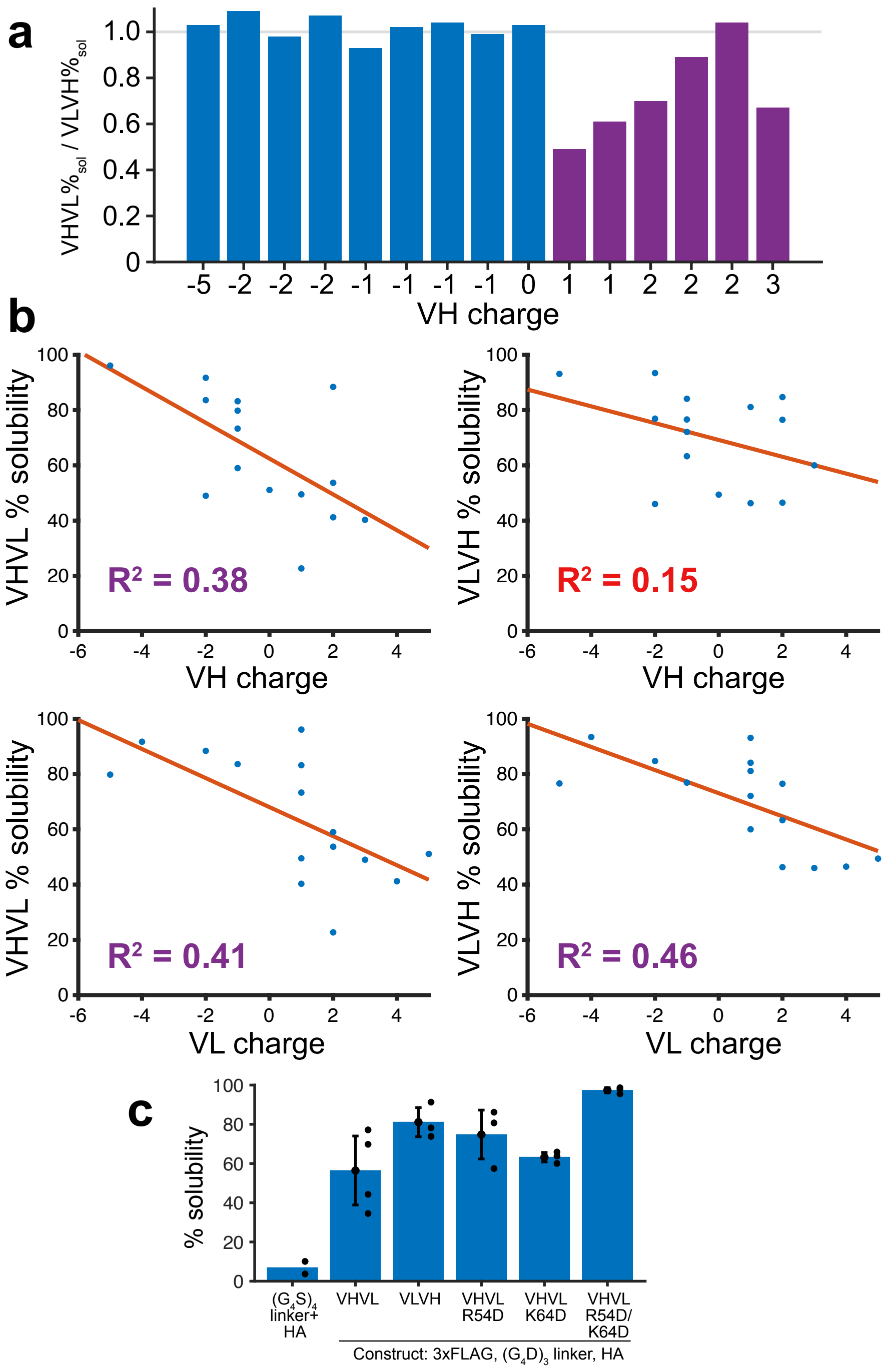

Supplementary Figure 3. Impact of variable heavy domain charge on scFv intrabody solubility. a. Ratio of VHVL/VLVH solubility for fifteen intrabodies with varying VH charge. **b.** Relationship between scFv intrabody variable domain charge and solubility of VHVL and VLVH constructs with coefficient of determination. **c.** Solubility of AMF7-63-derived anti-SOD1 scFv intrabodies and mutants. VHVL has lower solubility than the VLVH orientation, but this can be overcome by inclusion of charge swap mutations in the VH domain. N=2, 4, 3, 3, 3 biological repeats, $(G_4S)_4$/linker/HA vs VHVL R54D/K64D P=0.000056. Error bars represent one standard deviation. Chothia numbering. Source data are provided as a Source Data file.

Supplementary Tables

Region	Charge at pH 7.5 r^2 with solubility	Charge at pH 5.5 r^2 with solubility	Isoelectric point r^2 with solubility	Hydropathicity r^2 with solubility	Aliphatic index r^2 with solubility
**Fv (+ tags & linker)**	0.74	0.67	0.38	0.14	0.00
**VH**	0.09	0.07	0.08	0.00	0.01
**VL**	0.13	0.08	0.11	0.03	0.06
**All CDR**	0.14	0.10	0.09	0.03	0.01
**VH CDR**	0.04	0.03	0.07	0.03	0.00
**VH CDR1**	0.12	0.12	0.08	0.08	0.12
**VH CDR2**	0.05	0.05	0.08	0.01	0.05
**VH CDR3**	0.01	0.02	0.03	0.00	0.00
**VL CDR**	0.13	0.08	0.13	0.00	0.00
**VL CDR1**	0.09	0.08	0.15	0.00	0.00
**VL CDR2**	0.01	0.01	0.00	0.00	0.06
**VL CDR3**	0.13	0.03	0.05	0.01	0.05
**Framework Fv**	0.05	0.02	0.03	0.03	0.13
**Framework VH**	0.02	0.01	0.03	0.00	0.00
**Framework VL**	0.02	0.01	0.01	0.07	0.00

Property	r^2 with solubility
Mean pLDDT	0.00
Mean pLDDT*	0.07
Mean pLDDT#	0.05
DeltaG IF*	0.15
DeltaG IF#	0.07
DeltaG*	0.15
DeltaG#	0.07

*Whole molecule with aspartate linkers.
#Whole molecule with serine linkers.

Supplementary Table 1. Coefficient of determination (r^2) for predicted physicochemical and biophysical properties of scFv molecules broken down into regions of the molecule with experimentally determined solubility.

					Variable heavy				Variable light				Framework		
**Numbering scheme**	**Fv charge**	**VH charge**	**VL charge**	**All CDR**	**CDR 1 2 3**	**CDR 1**	**CDR 2**	**CDR 3**	**CDR 1 2 3**	**CDR 1**	**CDR 2**	**CDR 3**	**Fv**	**VH**	**VL**
IMGT	1.49	1.24	0.25	-1.57	-0.92	-0.16	-0.23	-0.52	-0.65	-0.03	-0.35	-0.27	3.05	2.15	0.90
Chothia	1.49	1.22	0.26	-1.41	-1.88	-0.14	-0.40	-1.34	0.46	0.45	0.29	-0.27	2.90	3.10	-0.20
Kabat	1.49	1.17	0.32	-0.74	-1.20	-0.14	0.28	-1.34	0.46	0.45	0.29	-0.27	2.22	2.37	-0.14

Supplementary Table 2. Comparison of variable domain CDR and framework mean charge of 8993 antibody-coding memory B cells[63] using IMGT, Chothia and Kabat amino acid numbering schemes.
