# Supplementary material for: Reliable repurposing of the antibody interactome inside the cell
Source: Nat Commun. 2026 Jan 31;17:2222. doi: 10.1038/s41467-026-69057-0 (PMC12963631; doi:10.1038/s41467-026-69057-0)
Supplement: Supplementary file 2 — Description of Additional Supplementary Files [file 41467_2026_69057_MOESM2_ESM.pdf]

Supplementary Data 1 – Sequence, description, predicted physicochemical characteristics and solubility of forty-five scFv intrabodies with various tags, linkers and domain orientations.

Supplementary Data 2 – 3B5H10-derived scFv intrabodies including sequence, construct ID, fusion tags, linker, net charge and ProteinMPNN$_{SOL}$ inverse folding settings**.**

Supplementary Data 3 – Sequence and description of 672 non-redundant scFv intrabodies targeting cytoplasmic proteins with native and inverse folded sequences.

Supplementary Data 4 – Anti-p53, UCHL1 and GFP scFv intrabody sequences with reference.

Supplementary Data 5 – Anti-SOD1 conformational specific scFv intrabodies with sequence and references.

Supplementary Data 6 – Anti-α-synuclein state specific scFv intrabodies with sequences and references.

Supplementary Software 1 – scFvright source code.
