# Supplementary material for: Reliable repurposing of the antibody interactome inside the cell
Source: Nat Commun. 2026 Jan 31;17:2222. doi: 10.1038/s41467-026-69057-0 (PMC12963631; doi:10.1038/s41467-026-69057-0)
Supplement: Supplementary file 9 — Supplementary Software 1 [file 41467_2026_69057_MOESM9_ESM.zip › Supplementary Software 1/index.php]

scFvright


# scFvright

## Predict scFv intrabody solubility from amino acid sequence

---

**scFvright predicts the percentage of a single chain variable fragment (scFv) found in the mammalian soluble cell fraction.**

**You can input paired variable domain, scFv with or without modifications and full-length antibody sequences.**

Load Example
php
$exampleFile = "/home/www/scfvright/Example.txt";
$exampledata = file\_exists($exampleFile) ? htmlspecialchars(file\_get\_contents($exampleFile)) : "";
?


**Select processing method:**

 Full-length. Best for scFv or variable domains. Includes tags and linkers etc.
  

 Variable domain only. Uses AbRSA to search for VH and VL. Tags, linkers, fusion proteins or non-variable domains removed.
  
  


**Paste your sequence data in FASTA format above, and hit the submit button below.**

Submit
Clear

**Depending on the number of sequences you submit, processing may take time (2000 sequences per minute).**

**Please get in touch if you would like help turning your antibody sequence data into soluble and functional intrabodies.**


---

**Confidentiality:** Sequences are confidential, not written to disk or saved.

---

Contact: gareth.wright@essex.ac.uk

University of Essex Accessibility Statement
