# Supplementary figures and images for: Reliable repurposing of the antibody interactome inside the cell
Source: Nat Commun. 2026 Jan 31;17:2222. doi: 10.1038/s41467-026-69057-0 (PMC12963631; doi:10.1038/s41467-026-69057-0)

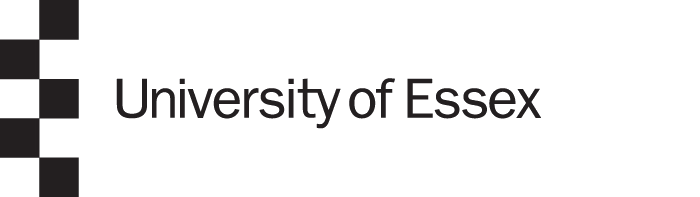

Supplement: Supplementary file 9 — Supplementary Software 1 [file 41467_2026_69057_MOESM9_ESM.zip › Supplementary Software 1/essex_logo.png]
